# Neural Cell Adhesion Molecule Ncam1b Promotes Effective Hair Cell Regeneration in Zebrafish Neuromasts

**DOI:** 10.3390/ijms27062738

**Published:** 2026-03-17

**Authors:** Annemarie Lange, Ramona Dries, Martin Bastmeyer, Joachim Bentrop

**Affiliations:** Cell and Neurobiology, Zoological Institute, Karlsruhe Institute of Technology (KIT), Fritz-Haber-Weg 4, 76131 Karlsruhe, Germany

**Keywords:** zebrafish, lateral line system, regeneration, differentiation, neural cell adhesion molecule

## Abstract

This study examines the distinct roles of the neural cell adhesion molecules Ncam1a and Ncam1b in zebrafish neuromasts during both homeostasis and hair cell regeneration. While both molecules contribute to the initial development of the lateral line system, previous work showed that a morpholino knockdown of *ncam1b* causes more severe developmental defects than *ncam1a* knockdown. However, in *ncam1b* mutants, only minor changes in FGF/Wnt signaling and cell proliferation are observed in the migrating primordium, which do not affect overall development of the lateral line development, suggesting compensation by Ncam1a. This work shows that after neomycin-induced hair cell loss, only Ncam1b is strongly re-expressed in regenerating hair and support cells. *ncam1b* mutants show delayed hair cell regeneration, with an increased number of proliferating support cells but impaired differentiation into hair cells. Notably, Ncam1a is not re-expressed during regeneration in *ncam1b* mutants. These regeneration defects likely arise from disrupted interactions of signaling pathways. Our data suggest that Ncam1b supports regeneration by sustaining the FGF pathway activity required for *atoh1a* induction. It also maintains balanced Notch signaling, which regulates support cell fate decisions. Together, these results highlight the crucial, non-redundant role of Ncam1b in coordinating signaling pathways to ensure proper hair cell regeneration in zebrafish neuromasts.

## 1. Introduction

Sensory hair cells in the auditory and vestibular systems are essential for hearing and balance in vertebrates. Homeostasis and survival of these cells are crucial for the lifelong function of such mechanosensory organs. Hair cells can, however, be damaged by various factors like infection, excessive noise, traumatic injury and age [1,2,3,4]. In mammals, cochlear hair cells in the mature inner ear lack significant spontaneous regenerative capacity, leading to permanent hearing loss after injury. While neurosensory hair cells in the vestibular organs retain a limited ability to regenerate even in adulthood, cochlear hair cells do not regenerate in adult mice or humans, with only transient and limited regenerative responses observed in neonatal rodents that are lost shortly after birth [5,6,7,8,9,10,11]. Non-mammalian vertebrates such as birds, reptiles, salamanders, sharks, and bony fish, by contrast, can regenerate hair cells throughout their lifetime [12,13,14,15,16,17,18].

In addition to the ear, zebrafish contain a second mechanosensory organ, the lateral line system, which plays a critical role in the detection of water movements. This structure is formed by a migrating primordium, a cluster of cells that deposit the sensory organs, so-called neuromasts, at regular intervals along the fish’s body. Neuromasts contain mechanosensory hair cells that are functionally and morphologically similar to those found in the mammalian inner ear, making the zebrafish lateral line an excellent model for studying hair cell development and regeneration [19,20,21,22,23,24]. Like mammalian hair cells, zebrafish hair cells are sensitive to damage from ototoxic drugs such as aminoglycoside antibiotics [25]. In contrast to mammals, zebrafish exhibit robust and rapid hair cell regeneration following noise- or drug-induced hair cell death, enabling direct investigation of cellular and molecular mechanisms underlying regenerative capacity. The ability to directly visualize lateral line hair cells and manipulate environmental exposure in concert with an array of genetic tools and high fecundity makes the zebrafish lateral line a useful system for understanding hair cell regeneration.

By 5 days post-fertilization (dpf), these hair cells are fully differentiated. This makes them sensitive to ototoxic agents like neomycin, which is commonly used to induce controlled hair cell ablation in regeneration studies [26]. Regeneration of the zebrafish lateral line system starts within 3–5 h after neomycin treatment, and hair cells are fully regenerated, both in form and function, within 2–3 days [27,28,29,30,31,32]. Hair cell regeneration in this system occurs primarily through two mechanisms, the proliferation of surrounding support cells followed by their subsequent differentiation into new hair cells, as well as direct transdifferentiation of support cells into hair cells. These processes are tightly regulated by pathways such as Notch, FGF and Wnt signaling, which balance cell division, differentiation, and tissue restoration [32,33,34,35,36]. Among these, the Notch pathway plays a pivotal role in determining support cell fate by maintaining them in an undifferentiated state and preventing premature differentiation into hair cells, thus ensuring proper tissue organization and regeneration [37].

We recently found that a morpholino-mediated knockdown of the gene encoding the neural cell adhesion molecule Ncam1b causes major defects in lateral line development [38]. In contrast, *ncam1b* mutants display only mild phenotypes, such as an altered spatial distribution of proliferating cells and changes in FGF and Wnt signaling in the primordium (primI) [39]. This discrepancy can be explained by partial compensation through the upregulation of *ncam1a* in the mutants. FGF signaling is moderately reduced in *ncam1b* mutants, likely because FGFR1a interacts less efficiently with Ncam1a. Despite this reduction, overall cell proliferation remains stable, suggesting that Ncam1b mainly controls where proliferating cells are positioned within primI.

Our present study was designed to extend the knowledge about Ncam1b function in the lateral line system beyond development. We find Ncam1b is also expressed in differentiated, functional neuromasts as well as during regeneration. Furthermore, during hair cell regeneration, neighboring support cells start to express Ncam1b. While *ncam1b* mutants display a reduced regenerative capacity of sensory hair cells, support cell proliferation is not impaired. Given the pivotal role of Notch signaling in maintaining support cells in an undifferentiated state [33,40], our finding that Notch inhibition rescues the phenotype in the *ncam1b* mutant implicates altered Notch activity as a contributing factor to the implied differentiation. This notion is further corroborated by ddPCR analysis showing changes in the expression of Notch target genes *atoh1a* and *her4*, along with downregulation of the FGF pathway target *etv4a*. These findings indicate that, during hair cell regeneration in zebrafish neuromasts, Ncam1b plays a pivotal role in mediating proper signaling through the FGF and Notch pathways, both of which are crucial for the accurate differentiation of support cells and the effective regeneration of hair cells.

## 2. Results

### 2.1. Ncam1b, but Not Ncam1a, Is Expressed in Neuromasts During Homeostasis

**Figure 1:**
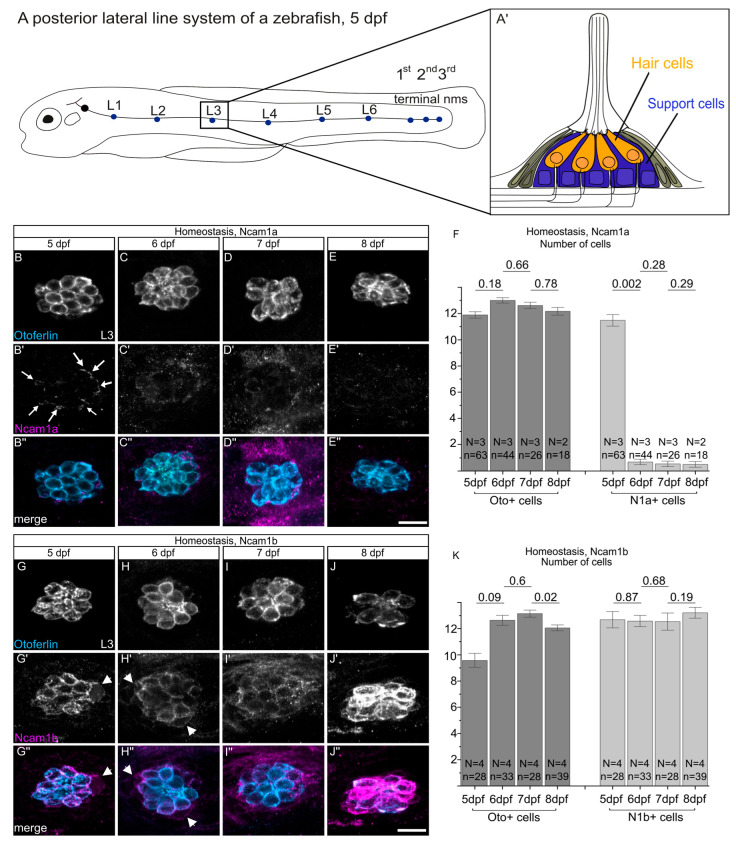
No expression of Ncam1a and strong expression of Ncam1b in hair cells during homeostasis. (**A**) Simplified illustration of the posterior lateral line system of a 5 dpf zebrafish, showing six primary neuromasts (L1–L6, blue dots) and three terminal neuromasts (blue dots) formed by primordium I (primI). (**A’**) Cross-sectional view of a neuromast, showing centrally located sensory hair cells (orange) surrounded by support cells (blue). (**B**–**E”**,**G**–**J”**) Confocal lateral views using laser scanning microscopy of the third primary neuromast (L3) in the transgenic zebrafish line *Tg(ClaudinB::lynGFP)* at different developmental stages (5–8 dpf). Rostral is to the left, caudal is to the right. (**B**–**E**) Immunohistochemical staining of Otoferlin (Oto+), a marker for immature as well as differentiated hair cells. (**B’**–**E’**) Immunohistochemical staining of Ncam1a. Note the weak expression in hair cells 5 dpf (white arrows). (**B”**–**E”**) Merged channels show the co-localization of Otoferlin (blue) and Ncam1a (magenta). (**G**–**J**): Immunohistochemical staining of Otoferlin (Oto+). (**G’**–**J’**) Immunohistochemical staining of Ncam1b, showing expression in hair cells and support cells (white arrowheads) at different stages. (**G”**–**J”**) Merged channels show the co-localization of Otoferlin (blue) and Ncam1b (magenta). (**F**,**K**) Quantification of Ncam1a- and Ncam1b-expressing cells during hair cell development. Bars indicate the median values of Otoferlin-positive (Oto+) and Ncam1a/Ncam1b-positive (N1a+/N1b+) cells in L3 from 5 dpf to 8 dpf, with error bars representing standard error. Two-sided two-sample *t*-test was used for statistics. Numbers above the data points represent *p*-values. Scale bars: 20 µm. Abbreviation: primI: first posterior lateral line system primordium; N: number of experiments; n: number of embryos; Oto+: Otoferlin-positive; N1a+: Ncam1a-positive; L3: third primary neuromast.

The current study was designed to elucidate a possible function of Ncam1a and Ncam1b in differentiated neuromasts and during hair cell regeneration. At 5 dpf, zebrafish have developed a branch of the posterior lateral line system derived from the first primordium (primI), comprising six deposited (L1–L6) and three terminal neuromasts (Figure 1A). Each neuromast contains centrally located sensory hair cells surrounded by support cells (Figure 1A’). We focused on 5 dpf larvae, a developmental stage at which neuromasts are fully formed and hair cells have attained functional maturity and susceptibility to ototoxic agents such as neomycin, thereby allowing their selective ablation in subsequent experiments [26]. Under homeostatic conditions, zebrafish neuromasts maintain a stable hair cell population (Figure 1B,B’), as reflected in the largely consistent number of Otoferlin-positive cells between 5 dpf and 8 dpf (Figure 1F,K; dark gray bars). Ncam1a only shows a transient expression pattern in hair cells of neuromasts. At 5 dpf, Ncam1a is weakly detected in the outer membrane of all hair cells (Figure 1B’ (white arrows) + F). The expression declines rapidly, both in intensity and in the number of expressing cells, until it is barely detectable by 6 dpf (Figure 1C’,F) and almost absent by 7 dpf to 8 dpf (Figure 1D’,E’,F). In contrast, Ncam1b shows sustained expression in hair cells from 5 dpf to 8 dpf (Figure 1G’–J’,K). Notably, this analysis is focused on the number of Ncam1b-expressing cells. Differences in expression intensity within individual cells were not assessed and are therefore not reflected in these data. These distinct expression patterns suggest that Ncam1b plays a more sustained role in differentiated neuromasts, while the function of Ncam1a appears to be limited to early developmental stages. This implies that Ncam1a functions mainly during the early stages of hair cell differentiation, while Ncam1b plays a more sustained role in maintaining differentiated neuromasts.

### 2.2. Ncam1b, but Not Ncam1a, Is Expressed in Hair Cells and Support Cells During Hair Cell Regeneration

**Figure 2:**
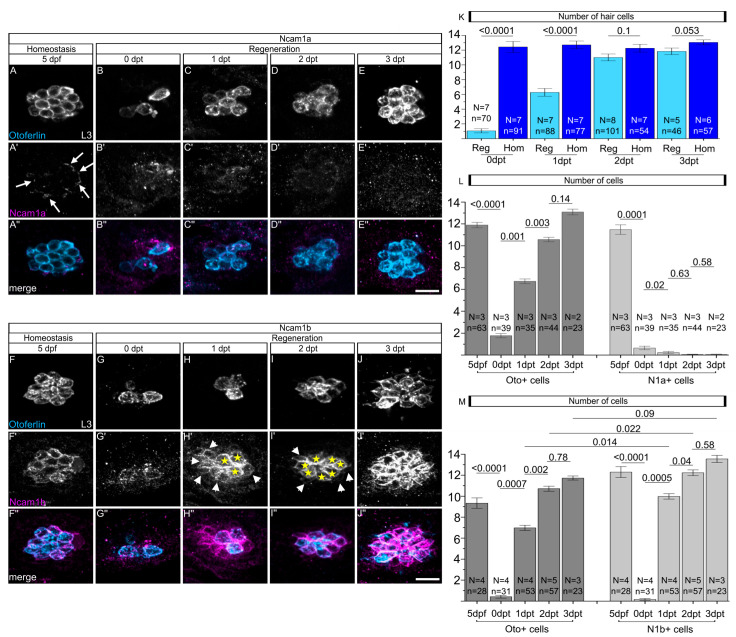
No expression of Ncam1a and strong expression of Ncam1b in hair cells during regeneration. (**A**–**E”**,**F**–**J”**) Confocal lateral views using laser scanning microscopy of the third primary neuromast (L3) in the transgenic zebrafish line *Tg(ClaudinB::lynGFP)* at different stages of hair cell regeneration (0 to 3 dpt). Untreated control embryos (5 dpf) are also shown. Rostral is to the left, caudal is to the right. (**A**–**E**) Immunohistochemical staining of Otoferlin (Oto+), showing the loss and regeneration of hair cells following neomycin treatment. (**A’**–**E’**) Immunohistochemical detection of Ncam1a. Note the lack of Ncam1a expression during regeneration. (**A”**–**E”**) Merged channels show the co-localization of Otoferlin (blue) and Ncam1a (magenta). (**F**–**J**) Immunohistochemical staining of Otoferlin (Oto+), demonstrating hair cell loss after neomycin treatment and subsequent regeneration. (**F’**–**J’**) Antibody detection of Ncam1b, which is expressed during regeneration. Yellow stars indicate Ncam1b expression in hair cells, white arrows indicate weak Ncam1b expression in the surrounding support cells. (**F”**–**J”**) Merged channels show the co-localization of Otoferlin (blue) and Ncam1b (magenta). (**K**–**M**) Quantification of Otoferlin-, Ncam1a- and Ncam1b-expressing cells during hair cell regeneration. The term 5 dpf represents the control group during homeostasis. To illustrate the homeostatic condition, the representative images shown for the 5 dpf control in panels (**A**–**A”**) and (**F**–**F”**) are the same as those presented in Figure 1. Likewise, the quantitative data for the homeostatic condition in panels (**K**–**M**) correspond to the datasets shown in Figure 1F,K. Bars indicate the median values with error bars representing standard errors. Two-sided two-sample *t*-test was used for statistics. Numbers above the data points represent *p*-values. Scale bars: 20 µm. Abbreviation: dpf: days post-fertilization; dpt: days post-treatment; N: number of experiments; n: number of embryos; Oto+: Otoferlin-positive; N1a+: Ncam1a-positive; L3: third primary neuromast; Reg: regeneration; Hom: homeostasis.

When damaged by neomycin treatment, sensory hair cells in zebrafish neuromasts regenerate efficiently through support cell proliferation and differentiation [25,31]. Given the expression of Ncam1a and Ncam1b during neuromast differentiation, we investigated whether these paralogs are functionally involved in hair cell regeneration. After neomycin treatment, nearly all functional hair cells in zebrafish neuromasts are ablated, except for some immature cells (Figure 2B,G,K). Regeneration and homeostasis are presented in two figures to enhance clarity. They were studied in the same experimental sessions to enable a direct comparison of cell numbers. The starting point (5 dpf) is thus generated from identical datasets (Figure 1B–B” and Figure 2A–A”, Figure 1G–G” and Figure 2F–F”, Figure 1F,K and Figure 2L,M, left columns). Quantification of Otoferlin-positive hair cells demonstrated robust regeneration, with full recovery to the levels of the untreated controls by 2 days post-treatment (dpt) (Figure 2B–E,G–K). While Ncam1a expression is observed in the outer membrane of homeostatic hair cells at 5 dpf (Figure 2A’, white arrows), the hair cells newly generated after ablation do not show Ncam1a expression (Figure 2B’–E’,L). In contrast, Ncam1b displays sustained expression throughout all phases of regeneration (Figure 2G’–J’,M). Notably, during the peak regeneration period (1 dpt and 2 dpt), the number of Ncam1b-positive cells significantly exceeds the number of Otoferlin-positive hair cells (1 dpt: *p* = 0.014; 2 dpt: *p* = 0.022) (Figure 2M). This indicates that, in addition to newly formed hair cells (Figure 2H’,I’, yellow stars), a considerable fraction of support cells re-expresses Ncam1b during regeneration (Figure 2H’,I’, white arrows, M). By 3 dpt, when regeneration is completed, Ncam1b expression becomes confined to hair cells (Figure 2J’,M). The broader, transient expression of Ncam1b in support cells during hair cell regeneration suggests a potential role for Ncam1b in the regenerative process.

### 2.3. ncam1b Mutants Exhibit Deficiencies in Hair Cell Regeneration

**Figure 3:**
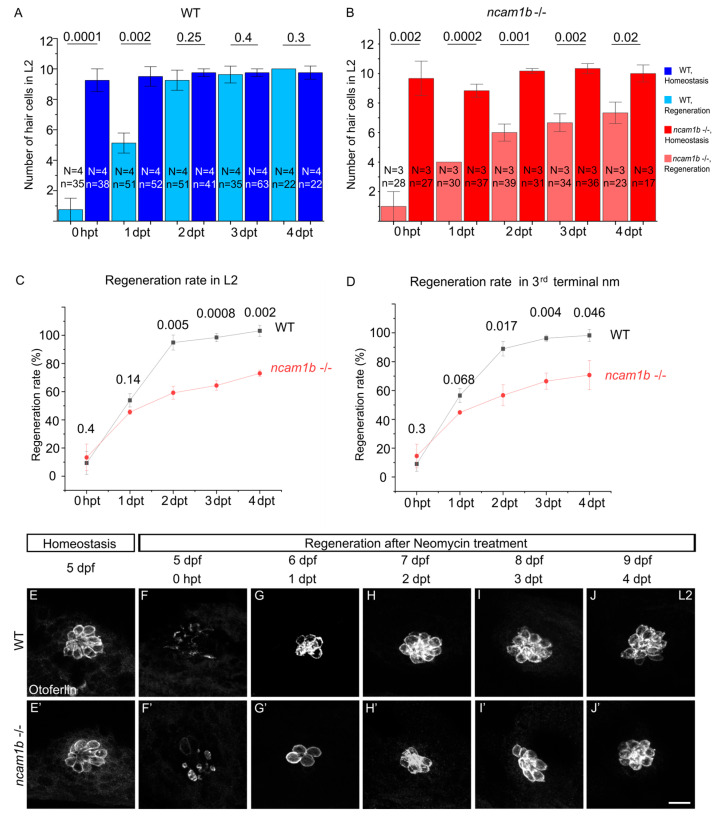
*ncam1b* knockout impairs hair cell regeneration. (**A**,**B**) Quantification of hair cells in neuromast L2 after neomycin treatment in wild-type (blue bars) and *ncam1b* mutant (red bars). The number of Otoferlin-positive cells in neomycin-treated embryos (light blue for wild-type and light red for *ncam1b -/-*) and untreated (homeostatic) embryos (dark blue for wild-type and dark red for *ncam1b -/-*) were counted and compared. (**A**) In wild-type embryos, hair cells are fully regenerated by 2 days post-treatment (dpt). (**B**) In *ncam1b* mutant embryos, hair cell regeneration is significantly reduced even at 4 dpt. (**C**,**D**) To determine the regeneration rate of hair cells, the number of newly formed hair cells after damage (at the respective time points following treatment) was calculated relative to the average number of hair cells present during the normal (homeostatic) state. The result was expressed as a percentage. L2 neuromast and the third terminal neuromast were investigated. (**E**–**J’**) Confocal images using laser scanning microscopy of hair cell regeneration in neuromast L2 in wild-type and *ncam1b* mutant embryos during (**E**,**E’**) homeostasis and (**F**–**J’**) following neomycin treatment. Otoferlin staining shows hair cells. Bars indicate the median values with error bars representing standard errors. Two-sided two-sample *t*-test was used for statistics. Numbers above the data points represent *p*-values. Scale bar: 10 µm. Abbreviation: dpf: days post-fertilization; dpt: days post-treatment; N: number of experiments; n: number of embryos; nm: neuromast; L2: second primary neuromast.

Following our observation that Ncam1b expression increases during regeneration, we next examined its role in this process utilizing a previously generated *ncam1b* mutant line [39]. Otoferlin staining reveals that both wildtype and *ncam1b* mutant zebrafish have similar numbers of hair cells in differentiated neuromasts between 5 dpf and 8 dpf (Figure S1), indicating that Ncam1b is not essential for the initial development of hair cells once proneuromast clusters have formed. We therefore concentrated on the function of Ncam1b during hair cell regeneration. To that end, we compared the regenerative capacity of wild-type and *ncam1b* mutant zebrafish following neomycin-induced hair cell ablation (Figure 3). Wild-type fish fully regenerate their hair cells by 2 dpt (Figure 3A,E–J), consistent with previous findings [27,41]. In contrast, *ncam1b* mutants display delayed regeneration (Figure 3B,E’–J’). The full number of hair cells is not yet reached by 4 dpt. To compare the regeneration efficiency, the number of newly formed hair cells at various time points after ablation was calculated as a proportion of the average number of hair cells present under normal (homeostatic) conditions (Figure 3C,D). At 1 dpt, we found no significant difference in the regeneration rate between wild-type and *ncam1b* mutants. By 2 dpt, however, *ncam1b* mutants exhibited markedly reduced regeneration efficiency, achieving only 73% of the regeneration rate observed in wild-type controls. This impairment persisted at 3 dpt and 4 dpt. These findings indicate that Ncam1b is important for efficient hair cell regeneration in zebrafish neuromasts. In summary, *ncam1b* mutants develop hair cells normally during development, yet exhibit impaired hair cell regeneration after damage.

### 2.4. No Ncam1a Upregulation During Hair Cell Regeneration in ncam1b Mutants

**Figure 4:**
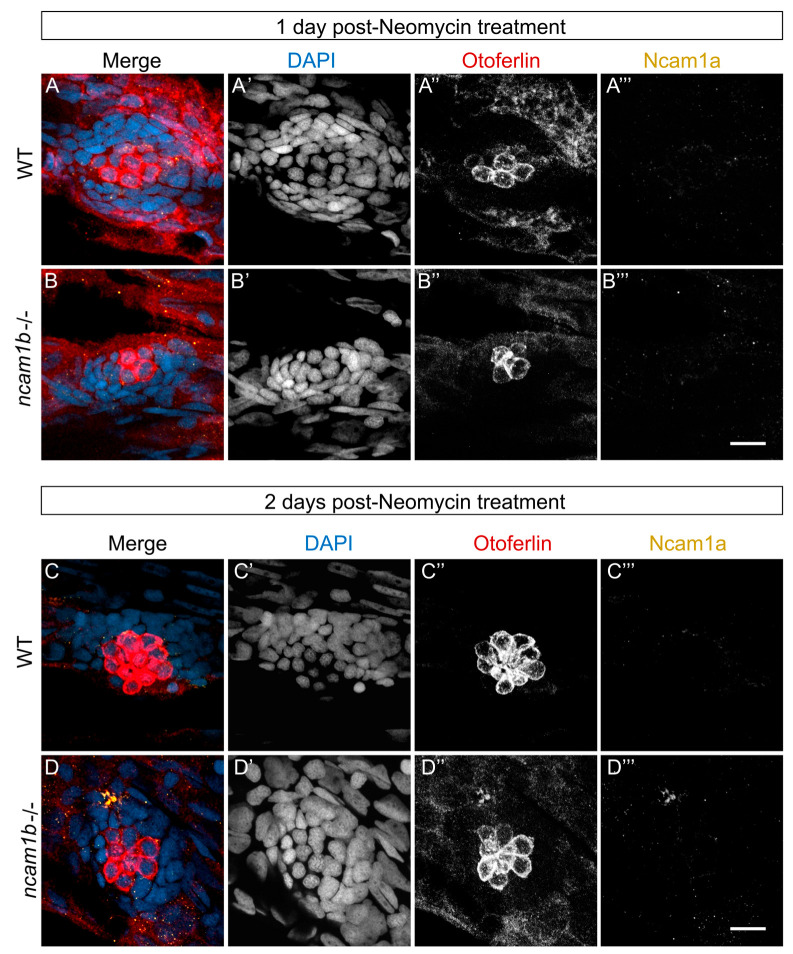
No Ncam1a upregulation in neuromasts during regeneration of hair cells, neither in wild-type nor in the *ncam1b* mutant. Confocal images using laser scanning microscopy of Ncam1a expression during hair cell regeneration in neuromast L2 were analyzed in (**A**–**A’’’**) 19 wild-type embryos (1 dpt), (**B**–**B’’’**) 16 *ncam1b* mutant embryos (1 dpt), (**C**–**C’’’**) 31 wild-type embryos (2 dpt), and (**D**–**D’’’**) 22 *ncam1b* mutant embryos (2 dpt) following neomycin treatment. Each condition was tested in three independent experimental replicates (N = 3). (**A’’**,**B’’**,**C’’**,**D’’**) Representative pictures for Ncam1a staining for each condition are shown. (**A’**–**D’**) Nuclei are stained with DAPI (blue). (**A’’**–**D’’**) Otoferlin staining shows hair cells (red). (**A’’’**–**D’’’**) Ncam1a (orange) is not expressed in wild-type and in *ncam1b* mutant neuromasts during hair cell regeneration at neither (**B’’’**) 1 day nor (**D’’’**) 2 days post-neomycin treatment. Scale bars 10 µm. Abbreviation: dpt: days post-treatment; WT: wild-type; L2: second primary neuromast.

The absence of Ncam1b does not completely block regeneration, suggesting that genetic compensation mechanisms might be active. Given the prior observation of *ncam1a* upregulation in *ncam1b* mutants during lateral line development [39], we tested whether a similar reparative mechanism might be activated during hair cell regeneration. To that end, we examined Ncam1a expression using immunohistochemical staining at two critical time points: 1 dpt, when regeneration rates between wild-type and *ncam1b* mutants are still comparable (Figure 4A–B’’’), and 2 dpt, when significant differences in regeneration efficiency first emerge (Figure 4C–D’’’). Surprisingly, despite sustained hair cell regeneration in *ncam1b* mutants, Ncam1a is not upregulated in the neuromasts at either time point (Figure 4B’’’,D’’’). Wild-type neuromasts show no upregulation of Ncam1a during regeneration as well (compare Figure 2 and Figure 4A’’’,C’’’).

### 2.5. Differentiation of Support Cells Is Impaired in ncam1b Mutants

**Figure 5:**
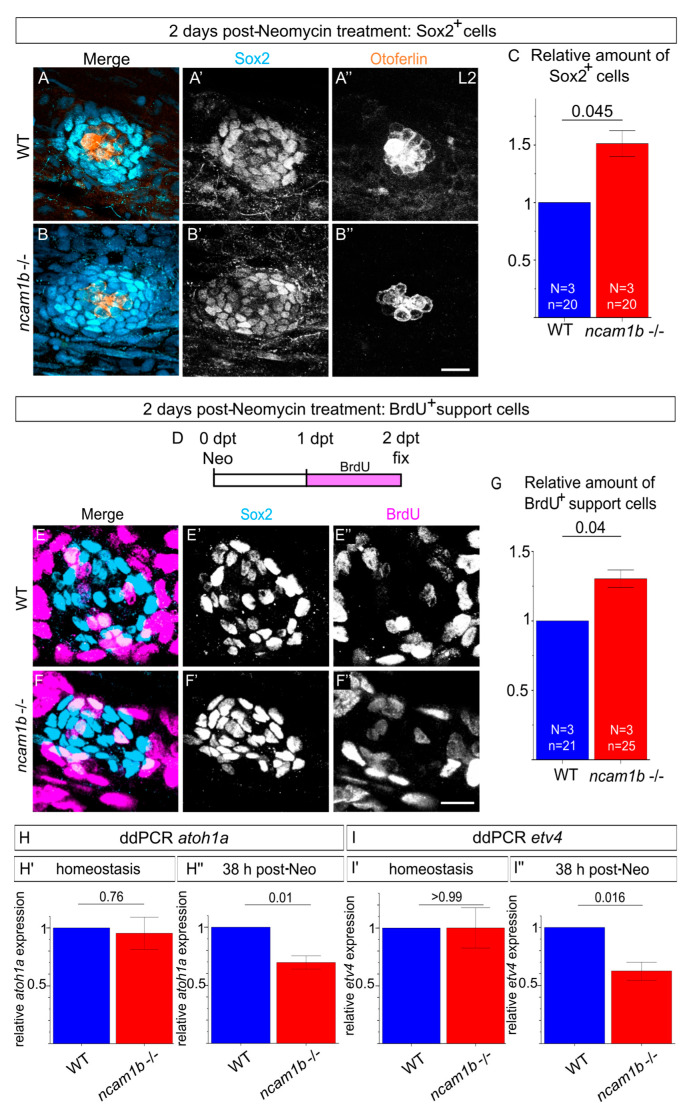
*ncam1b* mutants show an increase in proliferating Sox2-expressing cells during regeneration, accompanied by decreased expression of *atoh1a* and *etv4*. (**A**,**B**) Neuromasts L2 of wild-type and *ncam1b* mutant were stained with antibodies against Sox2 (light blue, (**A’**,**B’**)), Otoferlin (red, (**A”**,**B”**)) and with DAPI (not shown). Images were made using laser scanning microscopy. (**C**) Relative number of Sox2^+^ cells per neuromast (normalized to wild-type). Quantification shows that *ncam1b* mutants have more Sox2^+^ cells in neuromasts 2 dpt than the wild-type. (**D**–**G**) Embryos treated with neomycin were incubated with BrdU from 1 dpt to 2 dpt and then fixed with PFA. (**E**,**F**) Neuromasts L2 of wild-type and *ncam1b* mutant embryos were stained with an antibody against Sox2 (light blue, (**E’**,**F’**)) and with BrdU (magenta, (**E”**,**F”**)). Images were made using laser scanning microscopy. (**G**) Quantification of the relative number of BrdU^+^ support cells (Sox2^+^) (quantification as in C) shows that *ncam1b* mutants have more BrdU^+^ support cells in 2 dpt. Bars represent the mean of mutant values normalized to the corresponding wild-type values, with normalization based on the median gray value determined from each of three independent experiments. Error bars represent the standard error. Differences between wild-type and mutant were analyzed by two-tailed unpaired *t*-test with Welch’s correction. Numbers above the data points represent *p*-values. Scale bars 10 µm. (**H**,**I**) ddPCR analysis of *atoh1a* and *etv4* expression. Mean gene expression levels were determined using biological triplicates or quadruplicates, each representing the mean of technical duplicates. Bars represent the mean of mutant values normalized to the corresponding wild-type values. Differences between wild-type and mutant were analyzed by two-tailed unpaired *t*-test with Welch’s correction. Numbers above the data points represent *p*-values. Wild-type and *ncam1b* mutants were analyzed, with pools of 25 embryos per condition. (**H**) ddPCR analysis of *atoh1a* expression during homeostasis and regeneration at 38 hpt. (**H’**) Under homeostatic conditions, *atoh1a* expression did not differ significantly between wild-type and *ncam1b* mutants. (**H”**) Following neomycin treatment, *ncam1b* mutants displayed significantly lower *atoh1a* expression compared to treated wild-type siblings. (**I**) ddPCR analysis of *etv4* expression during homeostasis and regeneration at 38 hpt. (**I’**) Under homeostatic conditions, *etv4* expression did not differ significantly between wild-type and *ncam1b* mutants. (**I”**) Following neomycin treatment, *ncam1b* mutants displayed significantly lower *etv4* expression compared to treated wild-type siblings. Abbreviations: L2: second primary neuromast; WT: wild-type.

During regeneration in zebrafish neuromasts, support cells proliferate and differentiate into new hair cells. In *ncam1b* mutants, regeneration is retarded due to either reduced proliferation or impaired differentiation of support cells. Immunostaining for Sox2, a transcription factor that is essential for maintaining the support cell pool [42,43], revealed a significantly increased number of support cells (Figure 5A–C). In addition, BrdU assays showed higher numbers of BrdU-positive support cells in *ncam1b* mutants compared to wild-type (Figure 5E–G). Combined with reduced hair cell numbers, this indicates that Ncam1b is not essential to drive support cell proliferation but is critical for their differentiation into hair cells. To assess an impaired differentiation of support cells into hair cells, we compared *atoh1a* expression in wild-type and *ncam1b* mutants by ddPCR. *atoh1a* is a transcription factor critical for driving hair cell differentiation [44,45,46]. Our analysis was performed at 38 h post-treatment (hpt), a time point situated between 1 dpt—when hair cell numbers between genotypes are not yet significantly different—and 2 dpt—when regeneration is typically completed in wild-type (Figure 5H). During homeostasis, *atoh1a* expression at that time point was similar between wild-type and mutants, consistent with the similar number of hair cells under homeostatic conditions (Figure 5H’). In neomycin-treated *ncam1b* mutants, however, *atoh1a* expression was significantly lower than in neomycin-treated wild-type siblings (Figure 5H”), indicating a defect in the regenerative induction of *atoh1a* and suggesting impaired progression of hair cell differentiation in the mutant. Since *atoh1a* expression is tightly regulated by FGF signaling during regeneration, our findings raised the possibility that the reduction in *atoh1a* expression might result from impaired activation of the FGF pathway. To test this, we next examined the expression of the FGF target gene *etv4a* using ddPCR in wild-type and *ncam1b* mutants under homeostatic and regenerative conditions (Figure 5I). While no difference in *etv4* expression was observed between wild-type and *ncam1b* mutants under homeostatic conditions (Figure 5I’), a significant reduction in *etv4* expression was detected in the mutants during regeneration (Figure 5I’’), supporting the hypothesis of a regeneration-specific impairment of FGF signaling in the absence of *ncam1b*.

Together with the reduced *atoh1a* expression, which indicates an impaired activation of the differentiation program in support cells, these observations indicate that loss of *ncam1b* disrupts FGF signaling and thereby prevents support cells from differentiating into hair cells, keeping them in a proliferative, undifferentiated state.

### 2.6. Notch Inhibition Enhances Ncam1b Expression in Wild-Type and Rescues Hair Cell Regeneration Deficiencies in ncam1b Mutants

**Figure 6:**
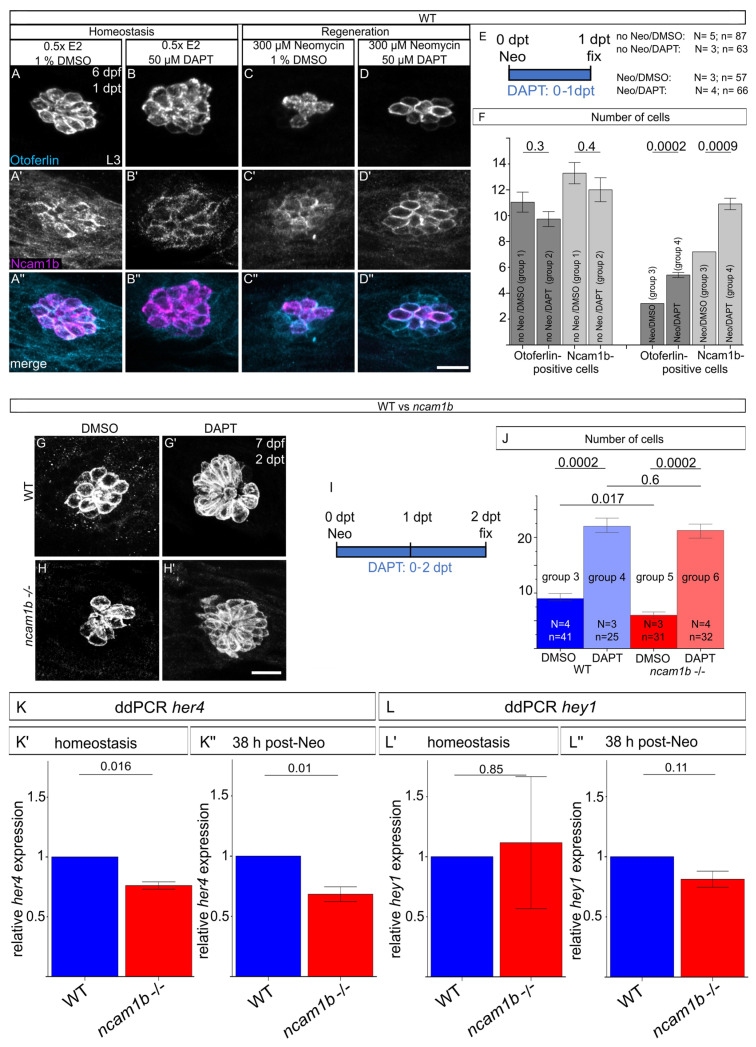
Notch inhibition impairs Ncam1b expression in wild-type, rescues hair cell regeneration defects in *ncam1b* mutants, and *ncam1b* mutants show reduced *her4* expression during homeostasis and regeneration. (**A**–**F**) Wild-type embryos were assigned to four treatment groups: (1) no neomycin + DMSO, (2) no neomycin + DAPT, (3) neomycin + DMSO, and (4) neomycin + DAPT. *ncam1b* mutants were analyzed in two additional groups: (5) neomycin + DMSO and (6) neomycin + DAPT. The condition “no Neomycin” was achieved by incubating embryos in 0.5× E2 medium. (**A**–**D”**) Confocal images of lateral views of neuromast were made using laser scanning microscopy. Rostral is to the left, and caudal is to the right. Notch inhibition in wild-type leads to significantly more Ncam1b-positive cells during regeneration. Neuromast L3 in wild-type zebrafish treated with DMSO or DAPT under homeostatic conditions (groups 1–2). Ncam1b immunostaining is shown in magenta and Otoferlin-staining is shown in blue. Note the increased Ncam1b expression in DAPT-treated fish after neomycin exposure (group 4). (**E**) Schematic of the experimental timeline for neomycin-induced hair cell damage and DAPT treatment (0–1 dpt) across wild-type groups 1–4. (**F**) Quantification of the number of Otoferlin-positive hair cells and Ncam1b-positive support cells in groups 1–4. (**G**–**J**) Notch inhibition rescues hair cell regeneration defects in the *ncam1b* mutants. (**G**–**H’**) Confocal images of neuromast L2 in wild-type (group 3 + 4) and *ncam1b* mutant zebrafish (group 5 + 6) during regeneration. Images were made using laser scanning microscopy. (**I**) Schematic representation of the experimental timeline for neomycin-induced hair cell damage and DAPT treatment (0–2 dpt) in wild-type and *ncam1b* mutants. (**J**) Quantification of the Otoferlin-positive hair cells in wild-type (group 3 + 4) and *ncam1b* mutants (group 5 + 6) shows that Notch inhibition rescues regeneration deficiencies in *ncam1b* mutants. Bars indicate mean values of the medians of each experiment, with error bars representing standard errors. Two-sided two-sample *t*-test was used for statistics. Numbers above the data points represent *p*-values. (**K**,**L**) ddPCR analysis of Notch target genes *her4* and *hey* in wild-type and *ncam1b* mutants (pools of 25 embryos per condition; biological triplicates or quadruplicates; each the mean of technical duplicates). Bars represent the mean of mutant values normalized to the corresponding wild-type values. Differences between wild-type and mutant were analyzed by two-tailed unpaired *t*-test with Welch’s correction. Numbers above the data points represent *p*-values. (**K**) *her4* expression during homeostasis and regeneration at 38 hpt. (**K’**–**K”**) *ncam1b* mutants show significantly reduced *her4* expression compared to treated wild-type siblings during both homeostasis and regeneration. (**L**) ddPCR analysis of *hey1* expression during homeostasis and regeneration at 38 hpt. (**L’**–**L”**) *hey1* expression does not differ significantly between wild-type and *ncam1b* mutants under either condition. Scale bars in (**D”**) 20 µm and in (**H’**) 10 µm. Abbreviation: N = number of experiments, n = number of embryos, L2: second primary neuromast, L3: third primary neuromast, WT: wild-type.

The upregulation of Ncam1b in support cells during hair cell regeneration suggests that these cells act as direct precursors of new hair cells. Since proliferation is not impaired in *ncam1b* mutants (Figure 5), our subsequent investigations focused on the differentiation process. Notch signaling, which regulates the balance between hair cells and support cells through lateral inhibition, was inhibited using DAPT, a gamma-secretase inhibitor [32,33,36]. Embryos were subjected to neomycin treatment or not treated with neomycin (homeostatic controls)—followed by treatment with either DAPT or DMSO during the same 1-day window post-neomycin (Figure 6A–F). This yielded four experimental wild-type groups: (1) no neomycin + DMSO, (2) no neomycin + DAPT, (3) neomycin + DMSO, and (4) neomycin + DAPT. Wild-type embryos in homeostatic conditions (groups 1 and 2) showed no differences in Otoferlin or Ncam1b immunostaining between DMSO- and DAPT-treated groups (Figure 6A–B’’,F, left). In contrast, DAPT treatment immediately after neomycin ablation (group 3 and 4) significantly increased the number of both Otoferlin-expressing hair cells and Ncam1b-expressing cells at 1 dpt (Figure 6C–D”,F). To elucidate a connection between Ncam1b and the Notch pathway during regeneration, *ncam1b* mutants were treated with DAPT until 2 dpt, the time point with the most pronounced difference in hair cell number between mutants and wild-type. This extended four wild-type groups to include two mutant groups: (5) *ncam1b* mutants + neomycin + DMSO and (6) *ncam1b* mutants + neomycin + DAPT. At 2 dpt, mutants (group 5) showed significantly fewer regenerated hair cells than wild-types (group 1; Figure 6G,H,J). DAPT treatment rescued this defect in mutants (group 6), yielding hair cell numbers comparable to the wild-type controls (group 2, Figure 6G’,H’,J). These results demonstrate that dysregulated Notch signaling underlies the regeneration defect in *ncam1b* mutants.

### 2.7. Disrupted Notch Signaling in ncam1b Mutants

Pharmacological inhibition of Notch signaling with DAPT indicates that dysregulation of the Notch pathway contributes to the regeneration defects in *ncam1b* mutants, pointing to altered Notch activity as a key underlying mechanism. To assess the contribution of Notch signaling to the regeneration defects in *ncam1b* mutants, we focused on the well-characterized Notch target genes *her4* and *hey1*, which are dynamically regulated during hair cell regeneration [36,40]. We performed ddPCR analysis of *her4* and *hey1* expression at 38 hpt, 10 h before wild-type is fully regenerated. No significant differences in *her4* and *hey1* expression were observed in wild-type or *ncam1b* mutants when comparing embryos in homeostasis and under regeneration conditions (38 hpt) (Figure S2). When comparing wild-type with *ncam1b* mutants during homeostasis, however, *her4* expression was significantly reduced in mutants (Figure 6K’), while *hey1* remained unchanged (Figure 6L’). During regeneration, as well, *her4* expression is significantly reduced in *ncam1b* mutants compared to the wild-type (Figure 6K’’). In contrast, *hey1* expression does not differ between *ncam1b* mutants and wild-type (Figure 6L’’). These results suggest that *ncam1b* modulates *her4* expression both under basal conditions and during regeneration, underscoring its role in regulating Notch signaling dynamics essential for hair cell regeneration.

## 3. Discussion

### 3.1. Distinct Expression of Ncam1a and Ncam1b in Lateral Line System Homeostasis and Regeneration

In zebrafish, *ncam1a* and *ncam1b* originated from a teleost-specific genome duplication event and show distinct expression patterns in mature neuromasts [38,39]. Ncam1a is transiently expressed in early proneuromasts and fades after 5 dpf, whereas Ncam1b persists in differentiated hair cells and a subset of support cells. During regeneration, Ncam1b is re-expressed in support cells and regenerating hair cells, where its persistent expression during peak regeneration (1–2 dpt) suggests a role in priming support cells for hair cell differentiation—consistent with the notion that neural programs are activated in support cells prior to the emergence of hair cell markers [31]. In contrast, the lack of Ncam1a in regenerating hair cells indicates that Ncam1a is largely dispensable for the regenerative process and may be restricted to early developmental differentiation events.

### 3.2. Ncam1b Has a Crucial Role During Hair Cell Regeneration at Later Stages

During development and homeostasis, Ncam1b is robustly expressed in wild-type neuromasts. *ncam1b* mutants, however, show normal hair cell number and no overt developmental defects, likely due to genetic compensation by *ncam1a* during development, as previously reported [39].

Our results demonstrate that wild-type larvae regenerate 95% of hair cells within two days after neomycin treatment, whereas *ncam1b* mutants reach only 73% even after four days. Early regeneration up to 1 dpt proceeds at a comparable rate in wild-type and mutants, suggesting that initial injury responses are largely Ncam1b-independent. In contrast, Ncam1b becomes essential for efficient progression through later regenerative phases. The partial regeneration observed in *ncam1b* mutants suggests that additional mechanisms can partially compensate for Ncam1b loss. Notably, Ncam1a upregulation does not occur during regeneration, indicating that compensation by the paralog is limited to development. Instead, yet unidentified molecules or parallel signaling pathways may support regeneration independently of Ncam1b. Candidate proteins include other cell adhesion molecules, such as L1cam, which has been implicated in promoting regenerative processes in the nervous system [47]. In summary, Ncam1b is dispensable for initial neuromast hair cell development but crucial for efficient late-stage regeneration. Similarly, in the *phoenix* mutant, support cells fail to regenerate due to misregulated proliferation, despite normal initial development [48]. These parallels demonstrate that genetic programs controlling support cell function during regeneration are distinct from those governing their original formation, indicating that regeneration is not merely a recapitulation of development but a process with unique molecular requirements.

### 3.3. The Absence of Ncam1b Leads to Accumulation of Proliferating Support Cells During Hair Cell Regeneration

Cell proliferation is a critical process in tissue regeneration, providing the necessary cell numbers to restore damaged tissues [49]. Loss of Ncam1b leads to an accumulation of proliferating Sox2-positive support cells that fail to differentiate into hair cells. Reduced expression of *atoh1a*—a key gene for neurogenic fate acquisition [40,50,51]—and a decreased nu mber of Otoferlin-positive hair cells indicate that progenitors are unable to efficiently initiate differentiation, remaining trapped in an undifferentiated state. This phenotype is consistent with the antagonistic relationship between Sox2 and Atoh1a, in which Sox2 normally maintains support cell identity and represses hair cell differentiation, whereas activation of Atoh1a suppresses Sox2 to drive hair cell formation [52]. In the absence of Atoh1a, however, progenitors maintain Sox2 expression and fail to differentiate.

### 3.4. Loss of Ncam1b Leads to Dysregulated FGF and Notch Signaling During Regeneration

During hair cell regeneration in the zebrafish lateral line, support cell fate decisions depend on a tightly balanced interplay between differentiation signals and inhibitory mechanisms. Our data position Ncam1b within this regulatory network.

We here report a reduced expression of the FGF downstream target *etv4* in the *ncam1b* mutant. Previous studies have already linked reduced or delayed FGF signaling, marked by lowered *etv4* expression, to defects in support cell differentiation [53]. The direct interaction between Ncam1b and the FGF receptor Fgfr1a stabilizes receptor signaling and promotes Erk phosphorylation independently of FGF ligands [38,54]. In the absence of Ncam1b, this stabilization is lost, potentially weakening Fgfr1a-mediated signaling, thereby reducing *atoh1a* expression, and ultimately blocking the differentiation cascade. Notably, the reduction in *etv4* expression was moderate, yet significant, indicating that FGF signaling is attenuated rather than being completely abolished. This suggests that Ncam1b does not function as an essential on/off component of the FGF pathway but instead fine-tunes the signaling amplitude and stability. Suboptimal FGF signaling in *ncam1b* mutants fails to induce *atoh1a* above the threshold required to induce the differentiation of hair cells.

Notch signaling also contributes to the *ncam1b* mutant phenotype. Pharmacological inhibition of Notch using DAPT restores the regeneration of hair cells to wild-type levels, indicating that Notch-dependent repression is a limiting factor for differentiation. Analysis of Notch target genes revealed complex regulatory interactions: *her4* expression was reduced in *ncam1b* mutants, while *hey1* remained unchanged. This indicates that Notch signaling is not globally down- or upregulated but shows target-specific dysregulation, likely reflecting altered feedback interactions or a context-dependent responsiveness of specific genes.

In the *ncam1b* mutant, reduced *her4* expression indicates diminished Notch activity, yet the residual signaling remains sufficient to prevent hair cell differentiation of neighboring cells. Due to attenuated FGF signaling, these cells express lower levels of *atoh1a*, which would otherwise promote hair cell formation, but the residual Notch activity is still capable of suppressing their differentiation. Consequently, support cells accumulate. Pharmacological Notch inhibition lowers the lateral inhibition threshold, allowing even the submaximal *atoh1a* expression to drive hair cell differentiation [45,46,55].

In summary, our findings identify Ncam1b as a novel component of the regulatory network governing hair cell regeneration through finely tuned signaling dynamics, underscoring the importance of signal thresholds in directing progenitor differentiation. Ncam1b stabilizes FGFR1a, thereby enhancing FGF/FGFR1a signaling and enabling sufficient induction of the proneural transcription factor *atoh1a*, which is essential to initiate hair cell differentiation. Within this framework, *atoh1a* acts as a pivotal integration node where pro-differentiation FGF signals and Notch-mediated lateral inhibition converge. In the absence of Ncam1b, FGF signaling is reduced but remains present; however, it fails to induce *atoh1a* expression to the levels required to overcome Notch-dependent repression. Notch activity is dysregulated in the absence of Ncam1b, further reinforcing the inhibition of hair cell differentiation and leading to an accumulation of *Sox2-positive* support cells.

## 4. Materials and Methods

### 4.1. Fish Strains and Animal Care

In our experiments, we used the transgenic fish strain *Tg(ClaudinB::lynGFP),* which was kindly given to us by Darren Gilmour (University of Zurich, Zurich, Switzerland) [56]. The *ncam1b* mutant zebrafish line is *ncam1b^ka902^*, a *ncam1b* -/- deletion mutant, generated in our lab [39]. This *ncam1b* -/- deletion mutant contains a large deletion of 205,908 base pairs, effectively removing nearly the entire gene, including its exons and introns, and introducing a stop codon in exon 1. Zebrafish were maintained under standard conditions at 28.5 °C in a ZebTec system (Tecniplast; Buguggiate, Italy). Embryos were kept in standard embryo medium (E3) at 28.5 °C for normal development. Embryos were allowed to hatch naturally. To prevent pigmentation, embryos were treated with 0.3% propylthiouracil (PTU; Sigma-Aldrich, St. Louis, MO, USA) starting at approximately 24 hpf. The E3 medium was changed daily, and fresh PTU was added until the day of fixation. Staging refers to Kimmel et al., 1995 [57].

### 4.2. Hair Cell Toxicity Assay

For hair cell ablation, 5 dpf larvae were incubated in 300 µM neomycin (Sigma-Aldrich, St. Louis, MO, USA) in 0.5× E2 embryo medium for 1 h and then washed three times in fresh embryo medium and then maintained in E3 medium at 28.5 °C. To analyze hair cell regeneration, embryos were fixed in 4% PFA in phosphate buffer before neomycin treatment, immediately after, or at 1 day post-treatment (dpt), 2 dpt, 3 dpt and 4 dpt. As a control, embryos were incubated with 1% DMSO in 0.5× E2 medium instead, representing the state of homeostasis.

### 4.3. Inhibition of Delta–Notch Signaling Pathway

To inhibit Notch signaling, embryos were transferred for 24 h or 48 h to a solution containing 50 µM DAPT in 0.5× E2 Medium at 28.5 °C. A control group was incubated in a solution of 1% DMSO in 0.5× E2 medium. Following treatment, the embryos were cooled on ice, fixed overnight in a 4% PFA solution in phosphate buffer at 4 °C, and then immunohistochemical-stained according to a standard protocol.

### 4.4. Immunostaining of Zebrafish Larvae

Immunostaining was performed following zebrafish standard procedures as previously described [58]. Ncam1a and Ncam1b were detected by rabbit anti-NCAM and anti-PCAM kindly provided by Yoshihiro Yoshihara (RIKEN Center for Brain Science, Saitama, Japan) (both 1:1000). In addition, mouse anti-HCS-1 (1:100; Developmental Studies Hybridoma Bank, Iowa City, IA, USA) was used for the staining of Otoferlin, and rabbit anti-Sox2 (1:300; GeneTex, Irvine, CA, USA) to stain support cells. To visualize proliferating cells, mouse anti-BrdU was used (mouse, 1:100; Abcam, Cambridge, UK). Embryos were subsequently placed in secondary antibody for 2 h at room temperature. For secondary antibody staining, Goat-α-Rabbit-Alexa488 (1:1000; Abcam; Cambridge, UK), Goat-α-Rabbit-Alexa647 (1:1000;Jackson ImmunoResearch Laboratories, West Grove, PA, USA), Goat-α-Mouse-cy3 (1:1000; Jackson ImmunoResearch Laboratories, West Grove, PA, USA), Goat-α-Mouse-Alexa647 (1:1000; Dianova; Hamburg, Germany) or Goat-α-Rabbit-Cy3 (1:1000; Dianova; Hamburg, Germany) was used. Mounting was performed in 20% Mowiol. For the determination of Ncam1- and HCS-1-positive cells in Figure 1 and Figure 2, the cell nuclei of the embryos were stained with DAPI; the respective single channels were overlaid with the DAPI channel (not shown) and analyzed. In other experiments, DAPI was sometimes not co-stained as the cells were still easily countable. In some cases, to identify neuromasts, either DAPI was used for counterstaining or the transgenic line *Tg(ClaudinB::lynGFP)* was used.

### 4.5. BrdU Assay

Proliferation was analyzed using bromodeoxyuridine (BrdU; Sigma-Aldrich; St. Louis, MO, USA). Embryos were incubated from 1 dpt to 2 dpt in 10 mM BrdU containing 1% DMSO at 28.5 °C. After rinsing with fresh E3 medium, embryos were fixed in 4% PFA in PBS at 4 °C for at least 4 h, and subsequently dehydrated in 100% methanol at −20 °C. Following fixation, DNA denaturation was performed using 2 N HCl for 60 min at 37 °C. Then, the embryos were stained with an antibody against BrdU to visualize proliferating cells. Only cells that showed clear BrdU staining in addition to Sox2 staining were evaluated as BrdU-positive support cells. To facilitate quantification and enhance clarity, Figure 5C,G present mutant-to-WT ratios calculated pairwise per experiment (N = 3). Within each experiment, the WT condition is normalized to 1, with mutant values expressed relative to this standard.

### 4.6. Droplet Digital PCR

Total RNA from pools of 25 whole-mount embryos was isolated using TRIzol and at least 500 ng RNA was used for reverse transcriptase using the SuperScript IV Reverse Transcriptase (Thermo Fisher Scientific, Waltham, MA, USA). An Automated Droplet Generator (Bio-Rad Laboratories, Hercules, CA, USA) was used to generate up to 20,000 reactions for ddPCR. The digital analysis of the individual droplets was performed using the QX200 Droplet Reader (Bio-Rad Laboratories, Hercules, CA, USA) and the associated QuantaSoft software (Version 1.7.4, Bio-Rad Laboratories, Hercules, CA, USA). Gene expression was normalized relative to *gapdh*, and then the expression was normalized to wild-type. All reactions were performed in technical duplicates; the results are the mean value of biological triplicates or quadruplicates. Primers: *gapdh*: forward: (5′-GTG GAG TCT ACT GGT GTC TTC-3′) and reverse (5′-GTG CAG GAG GCA TTG CTT ACA-3′); *ncam1a*: forward: (5′-GCACCGACCCTAAACCTACT-3′) and reverse (5′GGAATTCTTGGCAGGGTCAC-3′); *etv4*: forward: (5′-CAATGGAGAGCAGTGCCTTT-3′) and reverse (TTCATGGGGTAACTGTGGCT-3′); *hey1*: forward: (5′-GAGAAGGAGAGTGCGGATGA-3′) and reverse (5′-CCCTCTGCGACGTTTTCTT-3′); *atoh1a*: forward: (5′-GAGAGTTCTCGCCTCACTCG -3′) and reverse (5′- TCC GGC GGT GTG TTT TCT TA-3′); *her4*: forward: (5′-CGA ATC AAC AGC AGC ATC-3′) and reverse (5′-AAA TCA AGC GTC ATC TCC-3′). To facilitate quantification and enhance clarity, Figure 5H,I and Figure 6K,L present mutant-to-WT ratios from ddPCR measurements calculated pairwise per experiment (N = 3). Within each experiment, the WT condition is normalized to 1, with mutant values expressed relative to this standard.

### 4.7. Quantification and Statistical Analysis

Representative pictures in all figures were imaged using LSM800 laser scanning confocal microscope (Zeiss, Oberkochen, Germany). Images were processed and analyzed using the ZEN 2 lite software (blue edition) (Zeiss, Oberkochen, Germany) and the open-source FIJI distribution of ImageJ (version 1.54p). For the quantification of expression levels and the number of positive cells within individual neuromasts, a region of interest was created by manually outlining the neuromasts in the DAPI or CldnB channel. Within this region of interest, cells were subsequently counted and the fluorescence intensity was analyzed. Only cells that showed clear staining were evaluated as positive cells. Statistical analysis was performed using Excel and Origin 2019 (OriginLab Corporation, Northampton, MA, USA). For significance calculations, we utilized the *t*-test. For comparisons of relative values between wild-type and mutant (normalized to wild-type = 1), we utilized a two-tailed unpaired two-sample *t*-test with Welch’s correction. For comparisons of non-normalized data between two independent groups, a two-tailed unpaired two-sample *t*-test assuming equal variances was used. All tests were conducted at a significance level of α = 0.05. All diagrams were created using Origin 2019. Bar charts display the mean of the medians from individual experiments, with the whiskers representing the standard error.

## 5. Conclusions

In conclusion, Ncam1b is essential for neuromast hair cell regeneration, acting primarily to promote the differentiation of support cells into hair cells. In *ncam1b* mutants, support cells proliferate but fail to initiate the differentiation program, consistent with a primary role of Ncam1b in lineage commitment rather than proliferation. ddPCR analyses indicate reduced FGF signaling in the mutants, potentially reflecting a disrupted interaction between Ncam1b and Fgfr1a during regeneration. Impaired FGF activity likely contributes to the inability of support cells to acquire hair cell fate. Furthermore, evidence of dysregulated Notch signaling—manifested as reduced *her4* expression—and the rescue of the regeneration phenotype by DAPT treatment strongly implicate excessive or misregulated Notch activity in the mutant. Taken together, these findings identify Ncam1b as an important regulator that coordinates FGF and Notch signaling to enable proper support cell differentiation and effective hair cell regeneration in zebrafish neuromasts.

## Data Availability

The data presented in this study are available on request from the corresponding author.

## Supplementary Materials

The following supporting information can be downloaded at: https://www.mdpi.com/article/10.3390/ijms27062738/s1.

## Abbreviations

The following abbreviations are used in this manuscript:

dpf	days post-fertilization
dpt	days post-treatment
L1-L6	deposited neuromasts
Ncam	Neural cell adhesion molecule
primI	primordium I
