# Supplementary material for: Neural Cell Adhesion Molecule Ncam1b Promotes Effective Hair Cell Regeneration in Zebrafish Neuromasts
Source: Int J Mol Sci. 2026 Mar 17;27(6):2738. doi: 10.3390/ijms27062738 (PMC13026480; doi:10.3390/ijms27062738)
Supplement: Supplementary file 1 [file ijms-27-02738-s001.zip › ijms-4161089-supplementary.pdf]

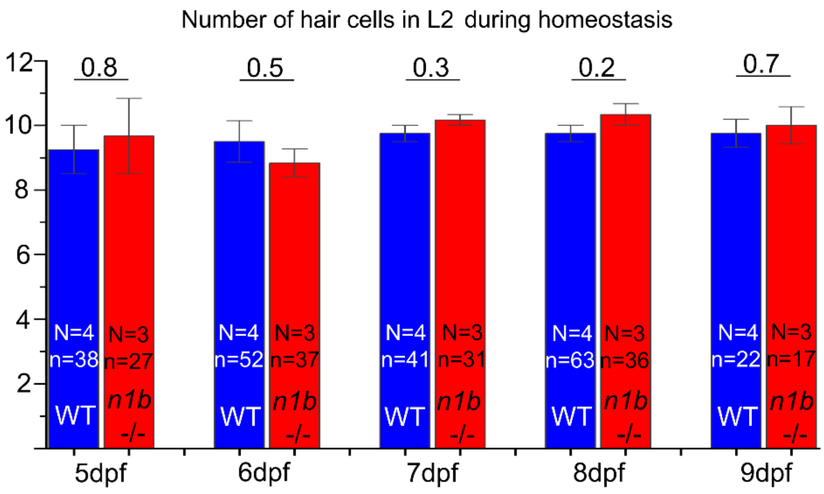

Figure S1: Similar number of hair cells in neuromast L2 of *wild type* and *ncam1b* mutant during homeostasis. Quantification of hair cell numbers in neuromast L2 of wild type (WT, blue bars) and *ncam1b* mutant (n1b -/-, red bars) zebrafish at 5–9 dpf. Hair cell counts were determined using immunolabeling against otoferlin. No significant differences (n.s.) were observed. Bars indicate mean values of the medians of each experiment, with error bars representing standard error. One sample *t*-test was used for statistics. Statistics were made using one sample *t*-test. *Abbreviation: L2: second primary neuromast; WT: wild type*

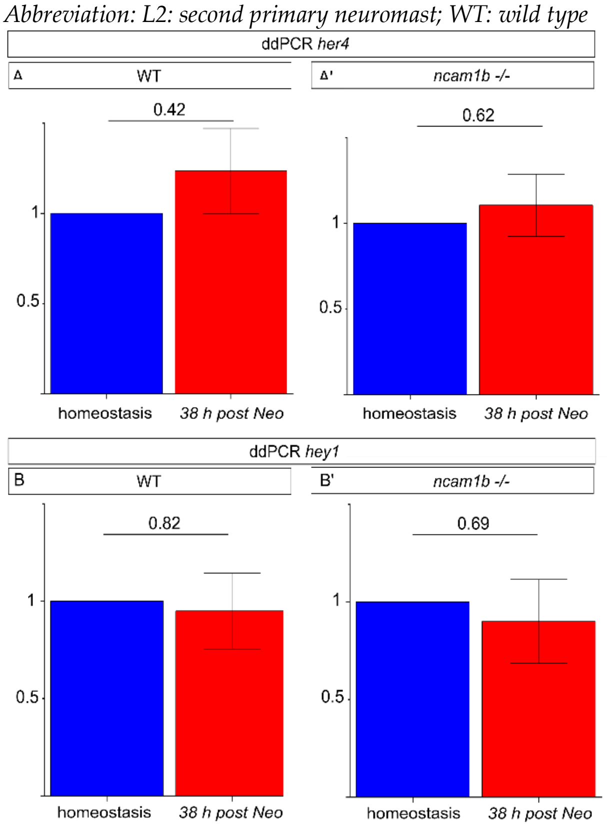

Figure S2: ddPCR analysis of Notch target genes *her4* and *hey*, under homeostatic conditions and 38 hours post neomycin treatment in wild type and *ncam1b* -/- mutants. Mean gene expression levels were determined using biological triplicates or quadruplicates, each representing the mean of technical duplicates. Wild type and *ncam1b* -/- mutants were analyzed, with pools of 25 embryos per condition. (A-A') No statistically significant differences in *her4* expression were observed between homeostasis and post-injury conditions in either (A) wild type and (A') *ncam1b* -/- mutant. (B-B') No significant differences in *hey1* expression were detected between homeostatic and post-neomycin conditions in either (B) wild type or (B') *ncam1b* -/- mutants. *Abbreviation: WT: wild type*
